# Study on the Hydration Reaction of Typical Clay Minerals under Alkali and Sulfate Compound Activation

**DOI:** 10.3390/gels8090564

**Published:** 2022-09-06

**Authors:** Siqi Zhang, Zeping Wu, Jiaming Chen, Runsheng Xu, Meina Wang, Wen Ni

**Affiliations:** 1 School of Civil and Resource Engineering, University of Science and Technology Beijing, Beijing 100083, China; 2 School of Metallurgical and Ecological Engineering, University of Science and Technology Beijing, Beijing 100083, China

**Keywords:** clay minerals, hydration product, compound activation, ettringite, calcium aluminate hydrate

## Abstract

Sand, stone, tailings and other aggregates often contain a small amount of clay mineral and their hydration activity is low, thereby lowering concrete performance indexes while negatively affecting their resource utilisation. In this study, clay minerals, calcium hydroxide and desulfurised gypsum were used to prepare cementitious materials to examine kaolinite, montmorillonite, illite and chlorite clay mineral contents under compound activation. The effects of curing temperature and water reducer on clay samples were analysed. The results showed that the compressive strength of kaolinite samples cured at 25 °C and 55 °C reached 1.09 and 4.93 MPa in 28 days and increased by 43% and 12%, respectively, after adding a 0.3% water reducer. Montmorillonite was activated and its compressive strength reached 5.33 MPa after curing at 55 °C in 28 days. Illite exhibited some activity and its compressive strength reached 1.43 MPa after curing at 55 °C in 28 days and the strength increased slightly after adding a water reducer. The chlorite sample had no strength after activation under the same conditions. Furthermore, X-ray diffraction and scanning electron microscope and energy-dispersive spectroscopy microstructure analyses showed that after alkali and sulfate activation, the hydration products of activated clay minerals mainly included ettringite, hydrated calcium aluminate and hydrated calcium silicate. The increase in curing temperature accelerated the reaction speed and improved the early strength. However, the effect on chlorite minerals was not obvious.

## 1. Introduction

Clay minerals, which are typically layered silicate minerals with a thickness of <2 μm, are a major component of soil. Their type and content greatly influence soil properties. Clay minerals are usually attached to the sandstone aggregate surface during their formation, excavation and crushing. Due to their layered structural characteristics and large specific surface areas, they have a certain adsorptive effect on the water reducer, which negatively affects various properties of concrete.

Studies have shown that clay minerals attached to aggregates, such as gravel and sand, infiltrate into the concrete system during concrete production, which reduces the workability [1,2], strength [3], deformation performance [4,5] and durability [6] of concrete to varying degrees. D. C. Pike revealed that increasing kaolinite, illite and montmorillonite content reduces concrete strength, with montmorillonite having a significant effect followed by illite and kaolinite [7]. M. A. Fam et al. suggested that clay minerals can adsorb cement hydration products, thereby affecting the hydration reaction of cement [8].

One of the most significant current directions in clay mineral usage is to stimulate the activity of clay minerals to make them become valuable components in the concrete system. D. Read et al. explored the long-term contact interface between cement and clay and found that a very thin transition layer appeared on the contact interface between the two. Through microstructure observation, the original structure of the two was damaged and new minerals were produced [9]. D. F. Noble et al. studied the reaction of clay cement mixture containing kaolinite, illite and montmorillonite and found only a small reduction of calcium hydroxide after long-term curing at room temperature; calcium hydroxide was also completely consumed after curing at 79 °C [10].

E. C. Gaucher et al. studied the transformation and dissolution kinetics of minerals in montmorillonite. Temperature and pH increase the dissolution rate, and the products include zeolite, hydrated calcium silicate and hydrated calcium aluminosilicate [11]. F. G. Bell et al. found that almost all clay minerals reacted with lime. When lime was added to the soil, clay minerals first reacted with Ca^2+^ at the bond breaking point under highly alkaline conditions (pH = 12.4) and the products included hydrated calcium silicate and aluminate [12]. Furthermore, E.C. Gaucher found that the reaction speed of clay minerals was slow under alkaline conditions (pH = 12, 13), but increasing the curing temperature to 150–200 °C favoured the reaction of clay minerals. The crystal structure of clay minerals has been shown to be seriously damaged under high temperatures [11].

Our previous research revealed that under the condition of compound activation by alkali and sulfate, aluminosilicate minerals with low hydration activity can be stimulated, mainly relying on the four environmental ions, Ca^2+^, SO_4_^2^^−^, OH^−^ and Al(OH)^−^, to form ettringite (3CaO·Al_2_O_3_·_3_CaSO_4_·32H_2_O) first. Owing to its small solubility, the formation of ettringite will continue to absorb the ions in the environment, thereby promoting further hydration reactions [13,14]. Xu Wang et al. used mineral micro powder to partially replace metakaolin to prepare a mineral micro powder metakaolin-based geopolymer under alkali activation. They found that the presence of calcium in mineral micro powder promoted the geopolymerisation reaction and at a powder content of 30 wt%, the polymer exhibited superior mechanical properties [15]. Mengya Niu et al. prepared a green and low-carbon geopolymer concrete using MFA as a partial replacement for metakaolin. The type of hydrated gel products was still a calcium silicoaluminate-based silicoaluminate gel in their study [16]. Xiaoyun Yang et al. explored the potential of using ultrafine calcined coal gangue and ground granulated blast furnace slag to develop a new geopolymer with the activation of a single activator (sodium hydroxide) or mixed activator (sodium hydroxide, liquid sodium silicate and desulfurization gypsum). They find the coal gangue geopolymers were mainly C–A–S–H, N–A–S–H and C–N–A–S–H gels and the mixed activator tended to yield higher strengths than the single activator because the hydration reaction was violent and produced more gels [17].

In this study, four typical clay minerals (kaolinite, montmorillonite, illite and chlorite), calcium hydroxide and desulfurised gypsum are used to prepare cementitious materials. Different curing temperatures and water reducer are selected as variable. Through X-ray diffraction (XRD), scanning electron microscope (SEM) and energy dispersive spectroscopy (EDS) analyses, the mineralogical changes and hydration reaction under composite activation conditions are studied.

## 2. Results and Discussion

### 2.1. Compressive Strength

**Figure 1:**
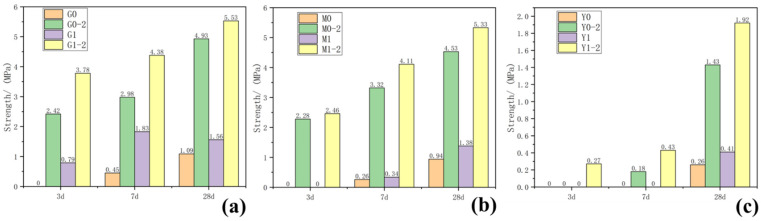
Results of 3-, 7- and 27-day compressive strength of samples: (**a**) group G, (**b**) group M, (**c**) group Y.

The compressive strength of each group of samples taken at 3, 7 and 28 days was tested, and the results are shown in Figure 1 and Table 1. As the curing age increased, the compressive strength of the samples increased to varying degrees. Increasing the curing temperature significantly promoted improved compressive strength. Adding a water-reducing agent to the control group samples with the same composition reduced the amount of water used in the sample preparation process to a certain extent, slightly lowered the water binder ratio and slightly increased the strength of the water-reducing agent group. As shown in Figure 1a, while the strength of G0 at 3 days, cured at 25 °C, was not measured, that of G0-2 at 3 days cured at 55 °C reached 2.42 MPa; the 3-days strength of G1 curing only reached 0.79 MPa, but that of G1-2 curing reached 3.78 MPa; after 28 days of curing, G0-2 strength was 4.93 MPa, which was ~352% higher than G0 strength (1.09 MPa). M0 and M1, cured at 25 °C for 3 days, had no strength and could only maintain the shape. However, after curing at 55 °C for 3 days, the strengths of M0-2 and M1-2 reached 2.48 and 2.26 MPa, respectively, and the strengths of 28 days curing reached 4.53 and 5.33 MPa, respectively. After curing at 55 °C for 3 days, the strength of Y1-2 added with the water reducer was 0.27 MPa. After 28 days of curing, the strength reached 1.92 MPa, which was 34% higher than that of Y0-2 after 28 days of curing. However, at curing temperatures of 25 °C and 55 °C or by adding a water-reducing agent, the chlorite samples did not generate strength in each test age.

### 2.2. XRD Phase Characteristics of Clay Samples

**Figure 2:**
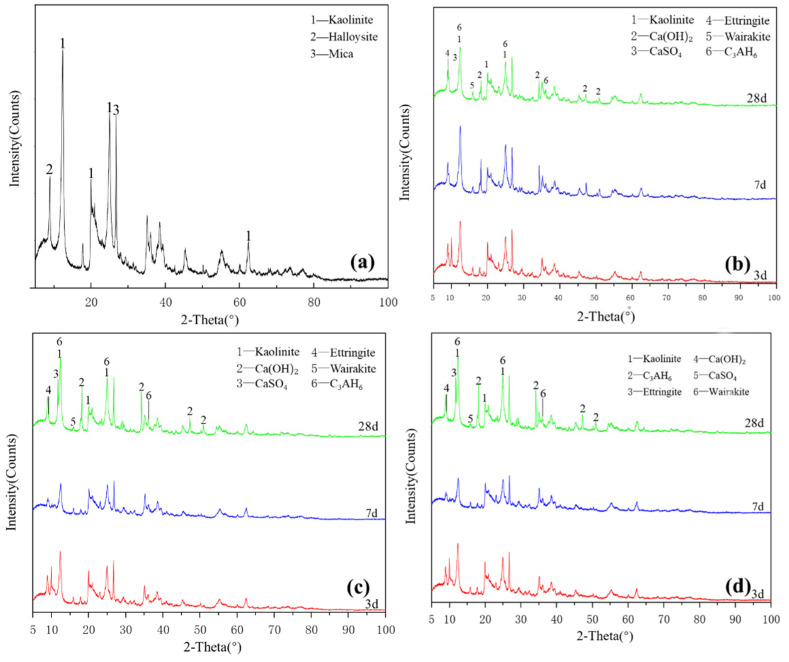
XRD patterns of kaolinite: (**a**) raw material, (**b**) 3-, 7- and 28-day curing age G0, (**c**) 3-, 7- and 28-day curing age G1, (**d**) 3-, 7- and 28-day curing age G1-2.

Figure 2a shows the XRD pattern of the kaolinite raw material. The main diffraction peak occurred at 2θ = 12.32°, 20.22° and 24.86° and the crystalline phase was mainly kaolinite, with a small amount of halloysite and mica. G0 samples are shown in Figure 2b. Under the condition of 25 °C curing, small amounts of monosulfide calcium aluminate hydrate, ettringite, clinoptilolite and calcium aluminate hydrate were generated in the G0 samples for 3 days. At 7 days, the peak of monosulfide calcium aluminate hydrate disappeared and the products were ettringite, calcium aluminate hydrate and clinoptilolite. After 28 days of curing, the peak strength of calcium hydroxide decreased. As shown in Figure 2c, under the condition of curing at 55 °C, a small amount of amorphous crystalline zeolite-like phase was found in the G0-2 sample of 3 days; the diffraction peak of calcium hydroxide disappeared after 7 days, which reappeared together with that of desulfurised gypsum after 28 days and the peak intensity of the product exceeded that of G0. Increasing the curing temperature in the early and middle stages of the reaction effectively accelerated the reaction speed of the kaolin group samples and the diffraction peak intensity of primary minerals decreased significantly. Figure 2d shows that the G1-2 sample was at diffraction peak 2 after adding a water reducer and curing at 55 °C for 3 days, the peak of monosulfide calcium aluminate hydrate at 9.8° and calcium aluminate hydrate formed a small ‘bulge’. After 7 days of curing, the diffraction peak intensity of ettringite and monosulfide calcium aluminate hydrate increased significantly and the characteristic peak of kaolinite decreased. After 28 days of curing, the diffraction peak of desulfurised gypsum did not appear in the G1-2 samples, the diffraction peak of calcium hydroxide was weak and the amount of ettringite increased slightly. The addition of a water-reducing agent did not significantly affect the reaction degree of kaolinite samples.

**Figure 3:**
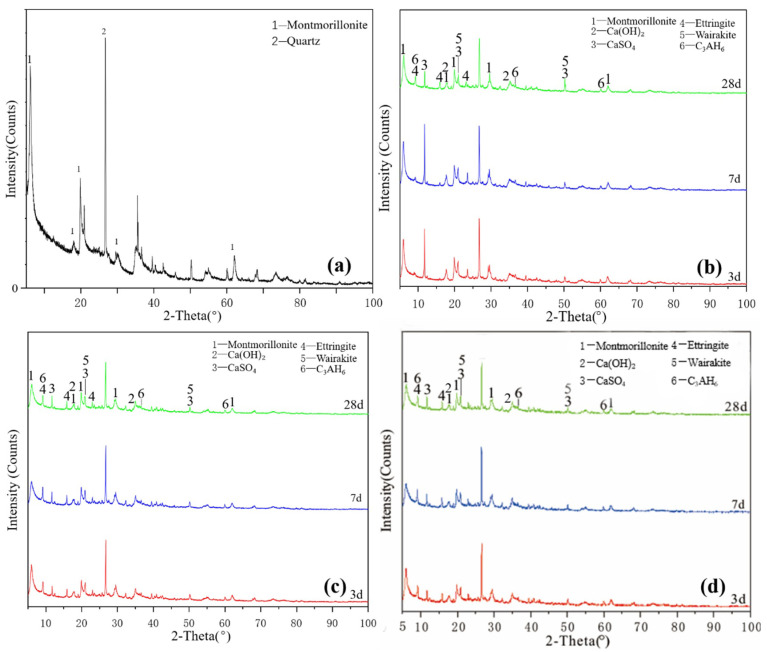
XRD patterns of montmorillonite: (**a**) raw material, (**b**) 3-, 7- and 28-day curing age M0, (**c**) 3-, 7- and 28-day curing age M1, (**d**) 3-, 7- and 28-day curing age M1-2.

Figure 3a shows the XRD pattern of the montmorillonite raw material. The main diffraction peak occurred at 2θ = 5.8°, 19.8° and 61.9° and the crystalline phase was mainly montmorillonite, with a small amount of quartz doping. Figure 3b shows that ettringite and hydrated calcium aluminate were generated in the M0 samples cured for 3 days. After 7 days of curing, a small amount of hydrated calcium aluminate and ettringite was generated, which probably contained orthophosphate zeolite. The diffraction peak intensity decreased after 28 days of curing, the reaction speed was relatively slow at 25 °C and the main products remained unchanged. As shown in Figure 3c, under the condition of curing at 55 °C, the diffraction peak intensity of M0-2 samples at 3, 7 and 28 days was basically the same. Increasing the temperature made montmorillonite consume much gypsum at the reaction’s early stage, thereby accelerating the early reaction degree. Adding a water reducer did not affect the raw material minerals and reaction products and only a small amount of it could improve the compressive strength (Figure 3d), which had no significant effect on the reaction speed and degree of montmorillonite in the system.

**Figure 4:**
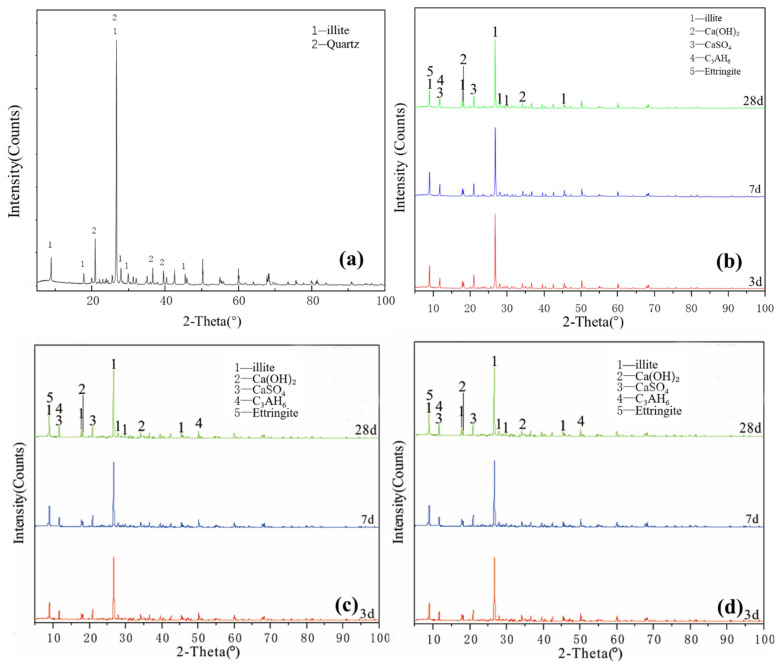
XRD patterns of illite: (**a**) raw material, (**b**) 3-, 7- and 28-day curing age Y0, (**c**) 3-, 7- and 28-day curing age Y1, (**d**) 3-, 7- and 28-day curing age Y1-2.

Figure 4a illustrates the XRD pattern of the illite raw material. The crystalline phase was mainly illite, with a small number of overlapping quartz phase peaks. The figure shows that a small amount of ettringite and hydrated calcium aluminate, except for unreacted desulfurised gypsum and calcium hydroxide, existed for the Y0 samples cured for 3 days. After 7 days of curing (Figure 4b), no new products were generated. After 28 days, except for the diffraction peak intensity of the existing products that slightly changed, no new products were generated and residues of desulfurised gypsum and calcium hydroxide remained. Y0 samples participated in the reaction less and the hydration reaction was slow. Under the condition of curing at 55 °C (Figure 4c), Y0-2 samples were cured for 3 and 7 days. In addition to calcium hydroxide and desulfurised gypsum peaks, a small amount of ettringite and hydrated calcium aluminate were newly produced. At 28 days, the characteristic peaks in the samples were similar and the intensity of the ettringite diffraction peak increased. Comparing the XRD patterns of Y0 at 28 days and Y0-2 at 3 days, the diffraction peak intensities of the reactants and products of both groups were equivalent, indicating that the reaction degrees of the two groups were similar. By adding a water reducer (Figure 4d), the characteristic peaks were basically the same, which only increased the compressive strength of illite samples to a certain extent; notably, increasing the curing temperature can induce improved compressive strength to a certain extent.

**Figure 5:**
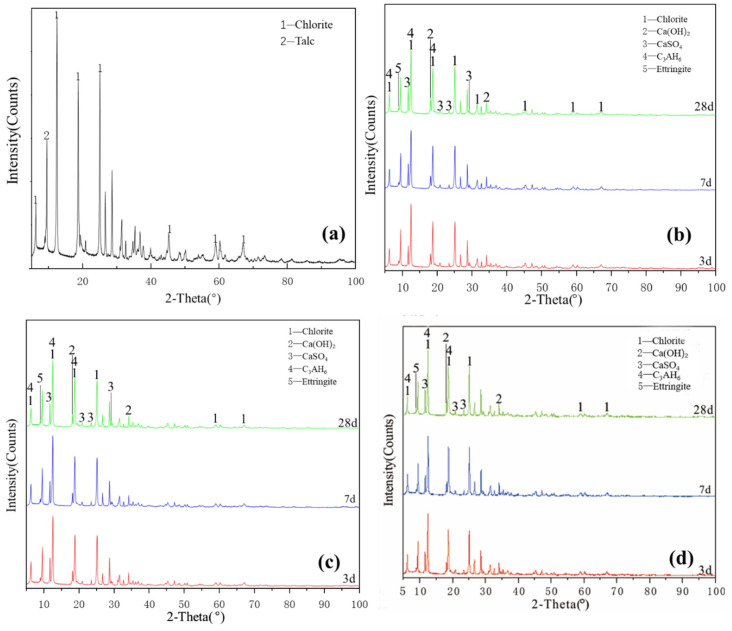
XRD patterns of chlorite: (**a**) raw material, (**b**) 3-, 7- and 28-day curing age Y0, (**c**) 3-, 7- and 28-day curing age Y1, (**d**) 3-, 7- and 28-day curing age Y1-2.

Figure 5a illustrates the XRD pattern of the chlorite raw material. The crystalline phase was mainly chlorite, with a small amount of talc crystalline phase peak. Figure 5a–c shows that the characteristic peaks of hydration products of L0, L0-2 and L1-2 at various ages slightly changed and the crystallisation degree of reaction products was poor. The new products formed probably included a small amount of ettringite, calcium aluminate hydrate, clinoptilolite and calcium silicate hydrate. The diffraction peak intensity of chlorite, talc, desulfurised gypsum and calcium hydroxide slightly decreased, indicating that chlorite did not participate in the reaction with desulfurised gypsum and calcium hydroxide; the paucity of the type and quantity of hydration products explained why the compressive strength of chlorite group samples was almost 0. After increasing the temperature and adding a water-reducing agent, the change in the degree of diffraction peak was extremely low and the sample was relatively stable; no obvious diffraction peak of reaction products was observed during the 28-day curing time. These findings showed that increasing the curing temperature does not significantly improve the reaction speed and degree of the chlorite system. The chlorite group samples were unable to harden during the curing process of 3–28 days, indicating that temperature and water reducer slightly affect the compressive strength of chlorite and desulfurised gypsum system and that chlorite cannot harden in this system for 28 days.

### 2.3. SEM–EDS Micromorphology Characterisation of Clay Samples

**Figure 6:**
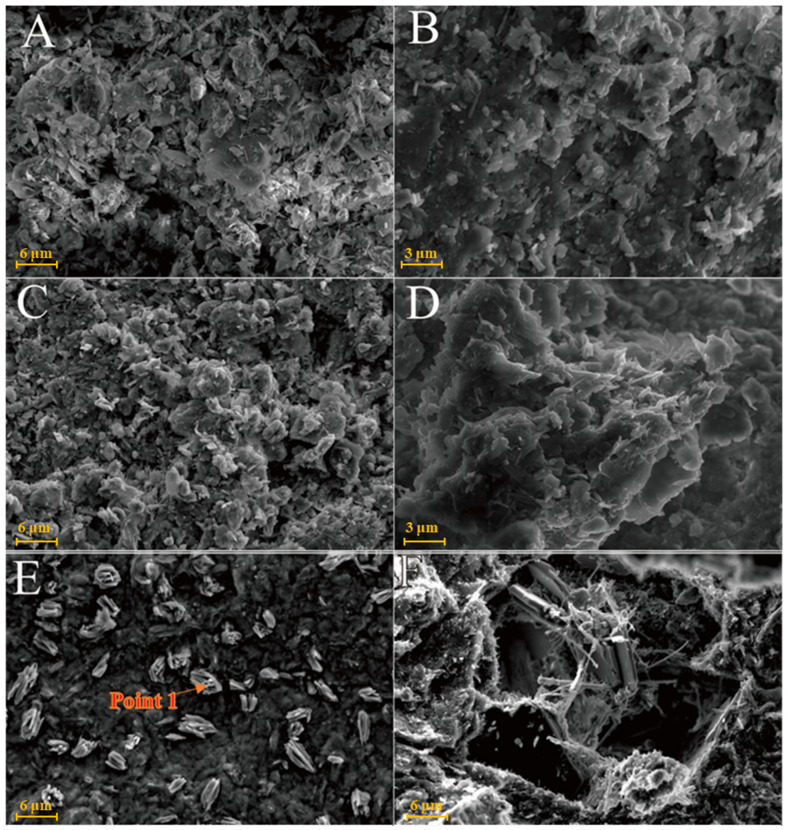

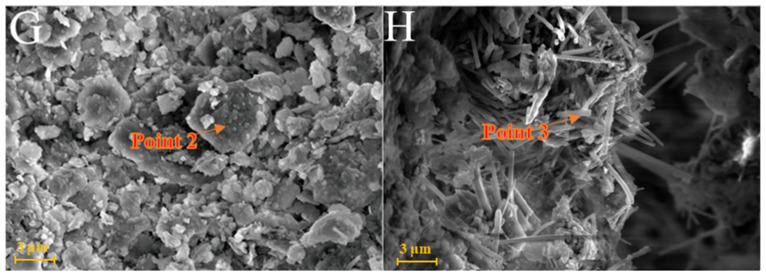
SEM images of kaolinite samples: (**A**)—kaolinite raw material; (**B**)—G0 samples cured for 3 days; (**C**)—G0 samples cured for 7 days; (**D**)—G0 samples cured for 28 days; (**E**)—G0-2 samples cured for 3 days; (**F**)—G0-2 samples cured for 7 days; (**G**)—G0-2 samples cured for 28 days; (**H**)—samples of G0-2 cured for 28 days.

As shown in Figure 6A, kaolinite raw materials are mainly irregular flakes under SEM and a single crystal size is 2–6 μm in the form of flakes and laminated. Acicular minerals are mainly halloysite and the crystal size is 1–3 μm. For the G0 sample cured for 3 days at 25 °C (Figure 6B), the generated gel-like substances bonded the particle, and some short columnar and acicular crystals were observed on the surface. Combined with EDS surface scan analysis, the crystals were monosulfide calcium aluminate hydrate. For the samples cured for 7 days (Figure 6C), spherical hydrated calcium aluminate appeared on the surface of the original mineral and a large number of amorphous gel-like substances were observed on the surface. Furthermore, a small amount of ettringite cluster aggregates with short crystals was observed in the samples cured for 28 days (Figure 6D). Three days after curing at 55 °C, a large number of hydration products were observed in the G0-2 samples, including a large number of cluster/cluster aggregates (Figure 6E). According to the EDS analysis, Table 2 shows ettringite. The microsurfaces of the 7-day sample were relatively dense, indicating that the reaction degree exceeded that of the G0 sample and the number of newly generated products was greater (Figure 6F). As shown in Figure 6G,H and Table 1, the surface of the G0-2 sample became very dense after curing for 28 days and a large number of ettringite appeared at the cracks and fractures. Compared with the samples of 7 days, the crystal size and number of ettringite increased significantly; ettringite appeared in clusters and columnar aggregates, with gel-like substances filling the gaps between the original minerals and the overall structure of the microsurface was further compacted. These findings showed that the kaolin samples had higher reaction degrees and reaction rates at high temperatures. As the curing time increased, the morphology of kaolinite crystals gradually disappeared, the crystal form of ettringite crystals gradually grew and thickened to form a frame structure and the hydrated calcium aluminate gel was filled in the gap to make the whole become dense. Increasing the curing temperature favoured this reaction and the number of reaction products significantly increased compared with that of G0, suggesting that increasing the reaction temperature can improve the reaction speed.

**Figure 7:**
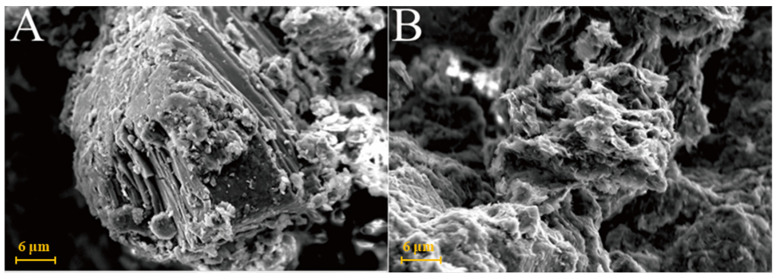

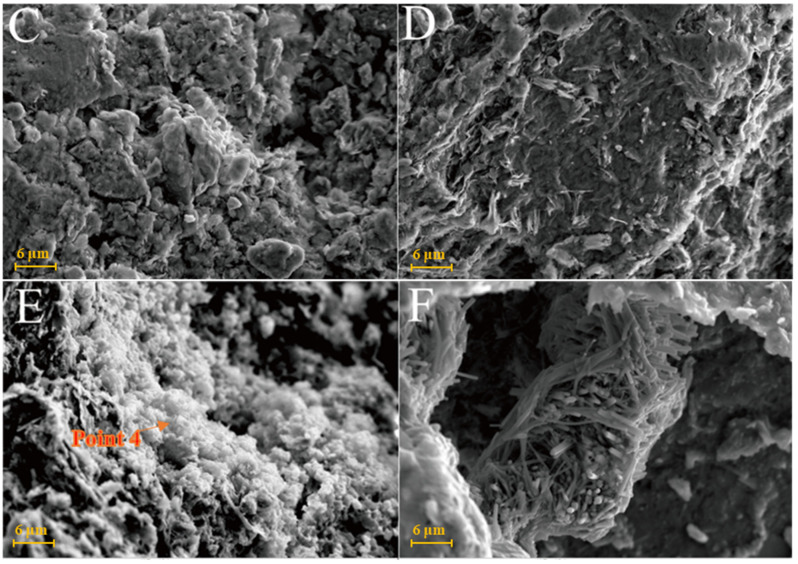
SEM images of montmorillonite samples: (**A**)—montmorillonite raw material; (**B**)—M0 samples cured for 3 days; (**C**)—M0 samples cured for 7 days; (**D**)—M0 samples cured for 28 days; (**E**)—M0-2 samples cured for 3 days; (**F**)—M0-2 samples cured for 7 days.

**Figure 8:**
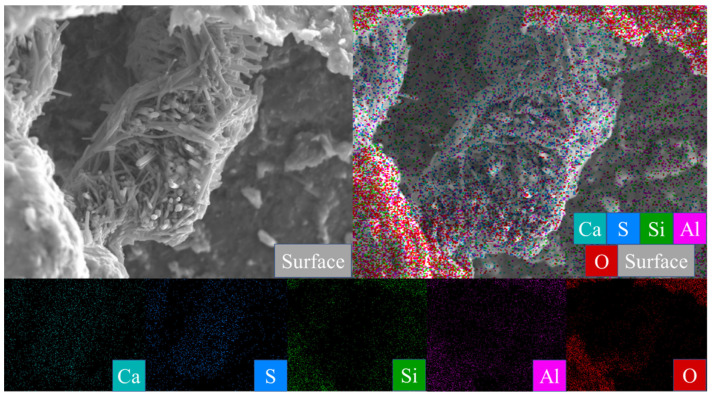
Scanning image of the M0-2 sample surface after 28 days of curing.

Figure 7A is the SEM pattern of montmorillonite raw material, showing laminated montmorillonite aggregates, lamellar montmorillonite crystals, honeycomb-mixed layers of Yimeng and other clastic minerals. When cured at 25 °C for 3 days, a small amount of gel-like crystalline substance was produced on the microsurface of M0 to cover the mineral surface (Figure 7B). Figure 7C,D show that the original form of montmorillonite gradually disappeared after 7 and 28 days of curing and the M0 sample was gradually compacted as a whole. Ettringite was observed in clusters or bundles, with crystals of about 1–2 μm or more. The shape of the original honeycomb-mixed layer was replaced by gel-like substances and granular gel aggregates existed on the microsurface. Under the condition of curing at 55 °C, a large number of granular gel aggregates were observed on the microsurface of the M0-2 sample for 3 days and short columnar crystal aggregates appeared in the cracks (Figure 7E and Table 2). After 28 days of curing, a large amount of ettringite was generated in the M0-2 samples (Figure 7F and Figure 8) and ettringite staggered to form a frame structure. The microsurface of the sample was very dense and the morphology of the original minerals was not found. Calcium silicate hydrate and calcium aluminate hydrate wrapped ettringite, which further improved the overall strength.

**Figure 9:**
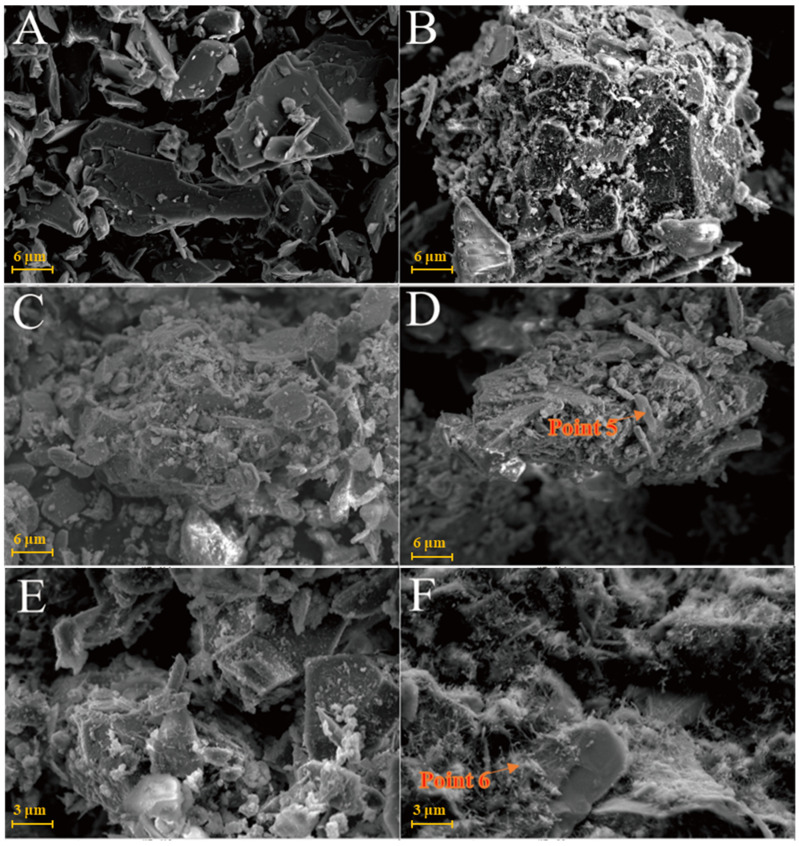
SEM images of illite samples: (**A**)—illite raw material; (**B**)—Y0 samples cured for 3 days; (**C**)—Y0 samples cured for 7 days; (**D**)—samples of Y0 cured for 28 days; (**E**)—Y0-2 samples cured for 3 days; (**F**)—Y0-2 samples cured for 3 days.

Figure 9A shows that the SEM images of illite raw material have a single crystal with a smooth surface and broken edges. In the Y0 samples cured at 25 °C for 3 days, small pieces of precipitates formed by the adhesion of illite could be seen (Figure 9B) and fine granular gel substances and needle crystals were observed on the illite surface. The morphology of illite crystals in the Y0 samples cured for 7 and 28 days could also be seen, with a rougher surface (Figure 9C,D). Furthermore, acicular ettringite and granular calcium hydrate aluminate covered the illite surface and the number of products significantly increased compared with that of the 3-day sample (Table 2). For the Y0-2 sample cured at 55 °C for 3 days (Figure 9E,F), granular hydrated calcium aluminate gel and needle-shaped ettringite could be observed on the surface of the sample attached to the illite surface and the gap between particles. Needle-shaped ettringite appeared in Y0-2 samples after 28 days of curing, growing on the surface like ‘hair’ and illite crystals were covered by ettringite and calcium aluminate hydrate gel. Ettringite crystals were fine and the number of ettringite crystals increased significantly as the curing temperature increased. As the amount of hydrated calcium aluminate is usually small, the overall compactness of the sample is poor.

**Figure 10:**
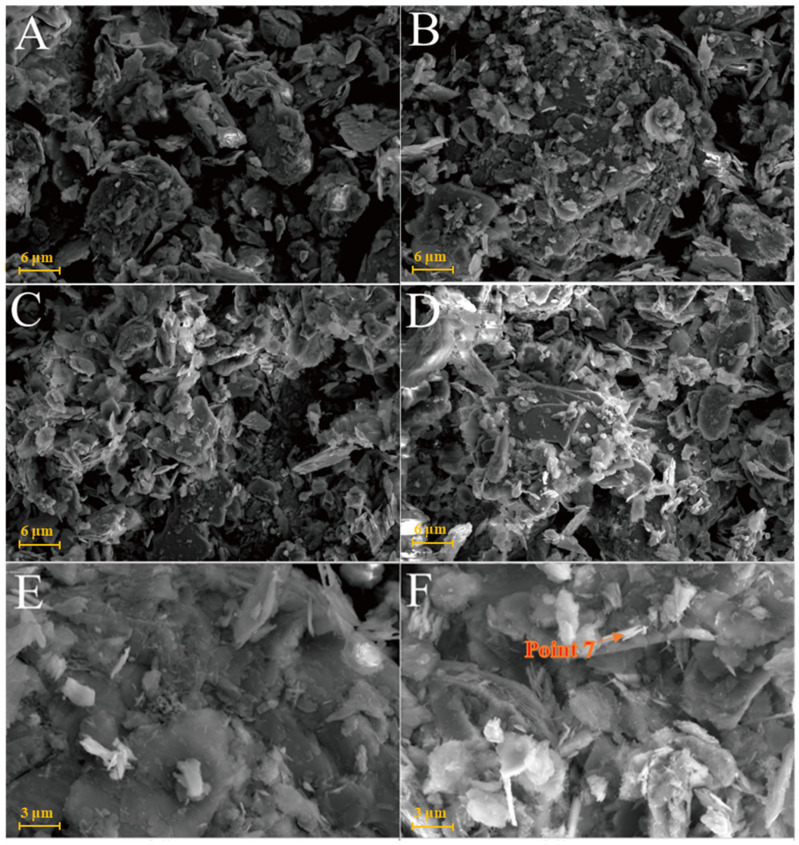
SEM images of chlorite samples: (**A**)—chlorite raw material; (**B**)—L0 samples cured for 3 days; (**C**)—L0 samples cured for 7 days; (**D**)—L0 samples cured for 28 days; (**E**)—L0-2 samples cured for 3 days; (**F**)—L0-2 sample cured for 28 days.

The chlorite raw material exhibited a broken sheet plate shape under SEM (Figure 10A). Gel and acicular ettringite were rarely observed on the surface of the L0 samples cured at 25 °C (Figure 10B–D). At 55 °C (Figure 10E,F), needle-like crystals and blocky gel with a large number of crystals on the surface were observed in the L0-2 samples after 3 days. After 28 days of curing (Table 2), the crystal form of chlorite itself remained complete and the whole was not dense. The formation of ettringite in the sample followed an increasing trend but remained in a fine needle-like shape. Chlorite was relatively stable in the system of desulfurised gypsum, calcium hydroxide and water. Although the curing temperature increased, the reaction speed did not significantly improve.

### 2.4. Discussion

The order of the compressive strength of the four clay mineral samples cured in the same en vironment for 28 days was kaolinite > montmorillonite > illite > chlorite, from large to small. The water-reducing agent promoted the compressive strength of kaolinite, montmorillonite and illite, without any effect on the compressive strength of the chlorite group. Raising the curing temperature significantly accelerated the reaction speed of kaolinite and montmorillonite and had a certain effect on illite without any effect on chlorite, primarily because the hydration ability of clay minerals differs due to the difference in the middle domain in the microstructure [18]. Notably, since the interlayer of montmorillonite is a van der Waals force (i.e., the interlayer force is weak) and its cation exchange capacity is high, montmorillonite has good hydration and high dispersion. The van der Waals force also exists between kaolinite layers and the hydration is good. The illite layer is K^+^ and the chlorite layer is a brucite flake. These two minerals have very weak hydration and poor cation exchange capacity.

As shown in Figure 2, Figure 3, Figure 4 and Figure 5, XRD was used to analyse the products generated by the reaction of four clay minerals with calcium hydroxide and desulfurised gypsum, mainly including ettringite, hydrated calcium aluminate [15,16,17]. A small amount of hydrated calcium silicate was produced in the montmorillonite group, which is consistent with the theoretical product inferred by P. Puscha et al.

The XRD data can be confirmed by SEM analysis of the micromorphology of the samples, but no calcium zeolite with a good crystal form was observed. The micromorphology of kaolinite and montmorillonite groups changed significantly. The morphology of the original mineral single crystal basically disappeared, ettringite formed a framework structure and calcium aluminate hydrate gel filled in the particle gap, making the whole become dense. The illite in the illite group also maintained its original form and the particles were bonded together by calcium aluminate hydrate gel, with ettringite forming a layer of needle-like crystals similar to ‘hair’ on the surface. The chlorite group only produced a small amount of ettringite crystals and gel on the surface and the chlorite itself did not change. These findings revealed that the strength source of group G and M samples was the joint action of gel, such as ettringite and hydrated calcium aluminate and that of illite group samples was acicular ettringite; meanwhile, no reaction was actually observed in the chlorite group samples, except for a small amount of ettringite that was formed, so strength was absent.

After adding water to the mixed raw materials, calcium hydroxide and desulfurised gypsum provided Ca^2+^, SO_4_^2−^ and OH^−^ for the system. Clay minerals underwent hydration and generated Al^3+^, Ca^2+^ and Mg^2+^ in a strong alkaline environment. In this system, Ca^2+^, Al^3+^, Ca^2+^, Mg^2+^, SO_4_^2−^, OH^−^ and Al(OH)^−^ were continuously consumed and combined to form reaction products, such as ettringite, calcium aluminate hydrate, calcium silicate hydrate and zeolite-like phase, which continuously reduced the number of the above ions and ion groups in the system [19,20,21]. Concurrently, silica tetrahedron and alumina tetrahedron or alumina coordination polyhedron with potential reaction in clay mineral structure were destroyed in the alkaline environment (in clay mineral structure, Si–O bond is the strongest, Al–O is slightly weaker, Mg–O is the second and Ca–O is the weakest), making ettringite and hydrated calcium silicate to form continuously. At the later stage of the reaction, the Si-Add 0.3% water reducer with total mass during the preparation of the four clay mineral samples, as calculated in Table 3. Except for group Y, the water-reducing rate of group G, M and L water reducers was only 10–12%, significantly lower than the nominal water-reducing rate of 25–30%. Compared with kaolinite, montmorillonite and illite samples with water reducers, the compressive strength of water reducer samples at the same age significantly improved, without any effect on the chlorite group. Adding a water-reducing agent to the sample could promote the system reaction to a certain extent while enhancing the dispersion effect of clay mineral particles and favouring the reaction to a certain extent. However, due to the large specific surface area of clay minerals, the water-reducing effect of a water-reducing agent will be greatly reduced, the O bond will be destroyed and active aluminium will be released to form hydrated calcium silicate gel. Increasing the curing temperature favours the acceleration of the reaction speed of the system; it only affects the number of reaction products and does not produce new products.

The hydration reaction in the clay mineral composite system is slow when there is only alkali excitation, but it is significantly accelerated by the addition of sulfate because the alkaline-activated of calcium hydroxide breaks the silica-aluminate mineral structure and promotes the dissolution of active silica-oxygen tetrahedra and alumina-oxygen tetrahedra [19,20], and then generates C–S–H gel (Equation (1)) and C–A–S–H gel (Equation (2)), while the desulfurisation gypsum plays a sulfate excitation role in the composite. The FGD gypsum plays a sulfate-activated role in the complex system [21], where the dissolved SO_4_^2−^ and Ca^2+^ continuously combine and react with the activated alumina tetrahedra to form calcium alumina [20]; see Equation (3), which forms the reaction driver and drives the hydration reaction of the whole system. Related studies [22] have shown that in the reaction system of slag, gypsum and cement rich in the glass phase of silica-alumina minerals, the equilibrium concentration of Aft and Al^3+^ or H_3_A1O_4_^−^ in solution is extremely low, which is much lower than the equilibrium concentration of Al^3+^ or H_3_A1O_4_^2−^ in solution of silica-alumina minerals, so that the continuous precipitation of calcium alumina drives the aluminum oxygen tetrahedra in silica-alumina minerals continuously. As a result, the precipitation of calcium alumina promotes the dissolution of aluminum-oxygen tetrahedra from silica-aluminate minerals, and the production of calcium alumina increases.

Ca(OH)_2_ + H_2_O + SiO_2_ = C–S–H (1)

Ca(OH)_2_ + (n − 1)H_2_O + Al_3_O_2_ + SiO_2_ = CaOSiO_2_·Al_2_O_3_·nH_2_O (C–A–S–H) (2)

3Ca(OH)_2_ + Al_2_O_3_ + 3(CaSO_4_·2H_2_O) + 23H_2_O = 3CaO·Al_2_O_3_·3CaSO_4_·32H_2_O (Ettringite) (3)

## 3. Conclusions

(1) The samples of group G, M and Y were cured at 25 °C for 28 days and the compressive strength values were 1.09, 0.94 and 0.26 MPa, respectively, which were 4.93, 4.53 and 1.43 MPa, respectively, after curing at 55 °C for 28 days. These values increased significantly by 12.17%, 17.66% and 34.27%, respectively, after adding a water reducer. Increasing curing temperature and adding water-reducing agent promoted the compressive strength of group G, M and Y samples, with no obvious effect on group L.
(2) XRD and SEM–EDS analyses showed that kaolinite, montmorillonite and illite reacted under the combined excitation of calcium hydroxide and desulfurised gypsum. The hydration products mainly included ettringite, hydrated calcium aluminate, hydrated calcium silicate and other gel. Increasing the curing temperature promoted the hydration reaction. Only a small amount of products were produced in the chlorite group samples after 28 days of curing and the hydration reaction was not obvious.
(3) Kaolinite, montmorillonite and chlorite had a significant impact on the water-reducing performance of high-performance polycarboxylate water reducer and the water-reducing efficiency was reduced from 25–30% to 10–13%. The water reduction rate of the polycarboxylic acid water reducer for the illite group samples reached 36.77%.
(4) The hydration activity of clay minerals is facilitated by using the compound activation method of alkali and sulfate, which can provide a basis for the utilisation of clay minerals in cementitious materials and concrete.

## 4. Materials and Methods

### 4.1. Preparation of Materials

Kaolinite, which was ground through a 200-mesh sieve, was selected from a kaolin factory in Shanxi Province. The chemical composition is shown in Table 4. The main components are SiO_2_ (56.34%) and Al_2_O_3_ (49.44%). The raw material of montmorillonite used in the study was produced in Xinyang, Henan Province, with a fineness of 1250 mesh. The chemical composition is shown in Table 4. The main components are SiO_2_ (69.15%) and Al_2_O_3_ (18.43%). The illite raw material was obtained from Hebei, with a fineness of 800 mesh. The chemical composition is shown in Table 4. The main components are SiO_2_ (73.22%) and Al_2_O_3_ (17.45%). The chlorite raw material was obtained from Hebei, with a fineness of 1250 mesh. The chemical composition is shown in Table 4. The main components are SiO_2_ (40.84%), MgO (38.45%) and Al_2_O_3_ (49.44%).

The desulfurised gypsum used in the study was the industrial solid waste desulfurised gypsum discharged from the wet desulfurisation of Chengde Iron and Steel group. The main component is basically similar to that of the natural desulfurised gypsum and the main mineral component is CaSO_4_·2H_2_O. The calcium hydroxide used was analytical pure calcium hydroxide produced by the Shanghai Sinopharm reagent group, with Ca(OH)_2_ more than 95%. The water reducer used was a polycarboxylic acid (PC) high-efficiency water reducer produced by Beijing Muhu admixture Co., Ltd. (Beijing, China).

### 4.2. Methods

This study followed a previous single-mineral research [23]. The internal environment of the slurry was set according to that of the hydration process of solid waste-based cementitious materials. When clay minerals (91%), calcium hydroxide (6%) and flue-gas gypsum (3%) were used as cementitious materials and the water binder ratio was 0.2–0.3, the four samples could not be demoulded in order to test their strength. When calcium hydroxide (10%) was added, the samples were still unable to demould and had no strength. Therefore, the content of calcium hydroxide and flue-gas gypsum increased simultaneously. Finally, clay minerals (83%), calcium hydroxide (10%) and flue-gas gypsum (7%) were used as cementitious materials that could be demoulded and tested for strength. The curing temperature and the dosage of the water reducer were changed to design comparative experiments. Table 5 lists the experimental proportions.

The slurry test blocks were made according to the standard of Portland cement and ordinary Portland cement (GB/T175-2007).

**Figure 11:**
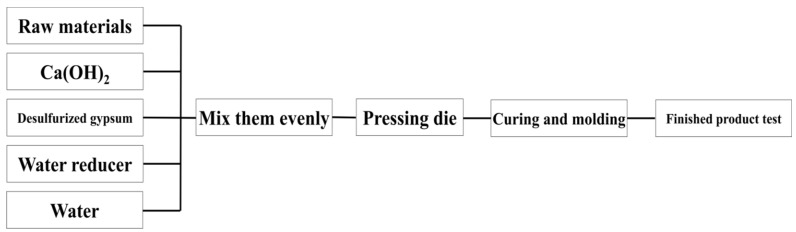
Flow chart of sample preparation of slurry test blocks.

As shown in Figure 11, the raw materials of kaolinite, montmorillonite, illite and chlorite were made into 30 × 30 × 50 mm test blocks with calcium hydroxide and desulfurised gypsum, respectively. The test blocks were grouped according to the curing temperature (25 °C/55 °C) and the experimental group with the same ratio was set to add an additional water reducer. The samples were placed in self-sealing bags to reduce the impact of water and carbon dioxide in the curing environment. Because a water reducer was added, the water binder ratio decreased slightly and the water reducer was 0.3% of the total mass of the experimental raw materials. After 3, 7 and 28 days of curing, the compressive strength of the test block was tested, respectively and the central part of the test block was soaked in absolute ethanol to remove the free water in the sample to terminate the sample’s hydration reaction.

### 4.3. Test and Analysis Method

(1) Compressive strength

The compressive strength measurements were performed according to the Test Method for Strength of Cement Mortar (ISO method) (GB/T17671—1999).

(2) Characterisation of samples

The mineral composition and crystallisation degree of raw materials and samples were characterised via XRD analysis (Japanese Neo-Confucianism Rigakuultima—Type IV powder crystal X-ray diffractometer, Cu target, wavelength 154.06 A, working current 40 mA, working voltage 40 kV, scanning range 5–90°, step size 0.02°).

A SEM was used to examine the micromorphology of raw minerals and the changes in new products and overall structure after the reaction. After carbon spraying on the sample surface, the sample was analysed using an SEM instrument (ZEISS SUPRA 55 field emission SEM). A Schottky field emission electron source was also adopted, which was equipped with an in-lens secondary electron detector, E–T secondary electron detector and 4Q BSD backscattered electron detector; resolution of X-ray energy spectrometer (EDS): 132eV@MnK α; detection element range: Be (4)~Fm (100); accelerating voltage: 0.1–30 kV.

## Figures and Tables

**Table 1:** Compressive strength of samples at different ages.

Samples	Water Reducer (g)	Curing Temperature (°C)	Three-Day	Seven-Day	Twenty-Eight-Day
			Compressive Strength (MPa)	Compressive Strength (MPa)	Compressive Strength (MPa)
G0 G0-2	0	25	—	0.45	1.09
	0	55	2.42	2.98	4.93
G1 G1-2	1.2	25	0.79	1.83	1.56
	1.2	55	3.78	4.38	5.53
M0	0	25	—	0.26	0.94
M0-2	0	55	2.48	3.32	4.53
M1	1.2	25	—	0.34	1.38
M1-2	1.2	55	2.26	4.11	5.33
Y0	0	25	—	—	0.26
Y0-2	0	55	—	0.18	1.43
Y1	1.2	25	—	—	0.41
Y1-2	1.2	55	0.27	0.43	1.92
L0	0	25	—	—	—
L0-2	0	55	—	—	—
L1	1.2	25	—	—	—
L1-2	1.2	55	—	—	—

**Table 2:** EDS dotting analysis of clay samples (Atomic percentage %).

Element	O	Al	Si	S	Ca
Point 1	58.62	10.64	1.22	10.29	19.24
Point 2	61.25	6.85	14.71	0.91	16.28
Point 3	30.93	13.19	0.86	15.87	39.15
Point 4	56.22	8.72	19.52	0.53	15.01
Point 5	57.87	9.58	22.04	4.08	6.43
Point 6	59.59	10.19	17.02	5.03	8.17
Point 7	64.20	5.83	19.08	4.82	6.07

**Table 3:** Water-reducing effect of water reducer on four clay mineral paste systems.

Group	G	M	Y	L
Reduce water consumption	21.80 g	15.80 g	61.30 g	20.00 g
Water reduction rate	11.37%	10.14%	36.77%	12.14%

**Table 4:** XRF analysis results of clay minerals used in this study.

	SiO_2_	Al_2_O_3_	K_2_O	Fe_2_O_3_	MgO	CaO	Na_2_O	TiO_2_	P_2_O_5_	MnO
Kaolinite	56.34	39.44	2.62	0.46	0.24	0.14	0.11	0.01	0.24	0.07
Montmorillonite	69.15	18.43	1.07	2.34	4.18	4.41	0.06	0.15	0.02	0.04
Illite	73.22	17.45	6.53	1.03	0.24	0.12	1.10	0.11	0.04	0.02
Chlorite	40.84	13.61	0.42	4.70	38.45	1.10	——	0.59	0.11	0.05

**Table 5:** Content of each sample component.

Group 25 °C/55 °C	Mineral Raw Materials (g)	Calcium Hydroxide (g)	Flue-Gas Gypsum (g)	Water (g)	Water Reducer (g)	Water Binder Ratio
G0/G0-2	332	40	28	191.8	0	0.48
G1/G1-2	332	40	28	170	1.2	0.43
M0/M0-2	332	40	28	155.8	0	0.39
M1/M1-2	332	40	28	140	1.2	0.35
Y0/Y0-2	332	40	28	166.7	0	0.42
Y1/Y1-2	332	40	28	105.4	1.2	0.26
L0/L0-2	332	40	28	164.8	0	0.41
L1/L1-2	332	40	28	144.8	1.2	0.36

Note. Mineral Raw Materials: G: Kaolinite; M: Montmorillonite; Y: Illite; L: Chlorite; 0 indicates the 25 °C curing temperature group and without water reducing agent; 1 indicates the group with water reducing agent addition; -2 indicates 55 °C curing temperature group.

## Data Availability

The data presented in this study are available from the corresponding author upon request.
